# Validating the Quality Maternal and Newborn Care Framework Index: A Global Tool for Quality‐of‐Care Evaluations

**DOI:** 10.1111/birt.12895

**Published:** 2024-11-11

**Authors:** Andrew Symon, Berit Mortensen, Are Hugo Pripp, Manju Chhugani, Samuel Adjorlolo, Caroline Badzi, Renu Kharb, Elysse Prussing, Alison McFadden, Nicola M. Gray, Allison Cummins

**Affiliations:** ^1^ School of Health Sciences University of Dundee Dundee UK; ^2^ Faculty of Health Sciences Oslo Metropolitan University Oslo Norway; ^3^ Jamia Hamdard New Delhi India; ^4^ University of Ghana Accra Ghana; ^5^ University of Newcastle, College of Health, Medicine and Wellbeing, School of Nursing and Midwifery Callaghan New South Wales Australia

**Keywords:** birth, labor, maternity care, postnatal, pregnancy, quality of care, survey

## Abstract

**Background:**

Quality maternity care is known to improve a range of maternal and neonatal outcomes. The Lancet Series on Midwifery's Quality Maternal and Newborn Care (QMNC) Framework is a high‐level synthesis of the global evidence on quality maternity care. Initial qualitative work demonstrated the Framework's adaptability in evaluating service user and provider perceptions of the quality of maternity care. However, evaluating services at scale requires a survey instrument. This paper reports the validation of the QMNC Framework index (QMNCFi), a five‐part survey for the evaluation of maternity care across diverse settings.

**Methods:**

International online English language survey of women who had given birth in the previous year in Australia, Ghana, India and the United Kingdom (UK). It was distributed through service user networks (UK and Australia) and at postnatal clinics (Ghana and India). All forms were completed online. Test–retest was conducted to assess reliability.

**Results:**

Five hundred and forty mothers completed the survey (Australia 136; Ghana 131; India 153; UK 120). Construct validity: Cronbach's *α* in 12 of the survey's 13 sections ranged from 0.795 to 0.986; for the remaining section the alpha was 0.594. Reliability: 55 participants completed the QMNCFi a second time. Intraclass correlation coefficient results ranged from 0.657 to 0.939 across the 13 sections. Field researchers in Ghana and India reported that the survey was easily understood and completed.

**Conclusion:**

This survey has shown that, across diverse contexts, the QMNCFi is a valid, reliable, and comprehensive tool for measuring service user perceptions of the quality of care over time.

## Graphical abstract

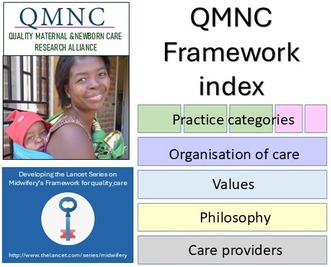

## 1 Introduction

Promoting quality maternity care is essential to foster positive outcomes for mothers and babies. However, despite some progress globally, the World Health Organization notes that maternal mortality rates are still “unacceptably high” [1], with most deaths occurring in low‐ and middle‐income countries. Focusing only on the biomedical causes of mortality has been criticized as an insufficient strategy for improving outcomes [2]. Substandard care can occur even in high‐income countries with regulated multidisciplinary maternity services including a developed midwifery profession, as evidenced by recent maternity care controversies in the United Kingdom (UK) [3, 4, 5] and the birth trauma inquiry in New South Wales, Australia [6]. There is good evidence that women around the world want respectful care and that their psychosocial well‐being is important [7].

Quality of maternity care is inextricably linked with clinical and psychosocial outcomes, including maternal mortality and stillbirth [8, 9, 10]. As detailed in a recent BMJ Collection [11], the World Health Organization and the World Bank have stressed that further attention and action are needed in order to promote and reinforce the improvement of quality maternal and newborn care in low‐ and middle‐income countries. Commissioners, providers, practitioners, and service users all need information on what constitutes quality maternity care, and on where quality care is deficient. The international drive to define and describe quality care led to a high‐level synthesis of the global evidence, culminating in the publication of the Quality Maternal and Newborn Care (QMNC) Framework [12] (“the Framework”) in the Lancet Series on Midwifery in 2014. This has influenced international benchmarks for antenatal care [13], national maternity care policy [14], and the UK's Standards of Proficiency for Midwives and Standards for Pre‐registration Midwifery Programmes [15, 16]. The International Confederation of Midwives' essential competencies for midwifery practice [17] have been mapped against the Framework's components, while the WHO has noted that these components “should be included in all activities to improve educational institutions, practice settings and clinical mentors” [18]. In 2019, the QMNC Research Alliance (www.qnmc.org) was launched to further the implementation of the Framework.

The QMNC Framework's comprehensive, global, and evidence‐based approach to identifying the components and characteristics of quality care, provides an effective benchmark for service evaluation across different service delivery contexts. The QMNC Framework was initially adapted for qualitative data collection in focus groups with service users and providers in the UK and Australia [19, 20, 21, 22]. This approach established that local care issues can be explored in depth using the Framework. However, a purely qualitative approach does not allow for measurement of specific aspects of care or for evaluations at scale. We therefore used a modified Delphi approach involving a 25‐person international panel, including several of the Lancet Series co‐authors as well as clinicians, researchers, and service user advocates to develop a survey version: the QMNC Framework index (QMNCFi) [23]. Early validation studies of the 85‐item QMNCFi in 2022 established face validity and promising psychometric properties; however, there were several redundant items. A revised 44‐item QMNCFi consisting of five domains was developed. This paper reports the results of a global multicountry validation and reliability study of this revised, shorter, version of the QMNCFi.

## 2 Methods

We conducted a validation study involving an international online survey of women who had given birth in the last year and who had received formal (i.e., professionally delivered) maternity care during pregnancy and/or labor/birth and/or the postnatal period. Participants in Australia, Ghana, India, and the UK were included if they were ≥ 18 years (16 in Scotland) and had access to the internet and were able to understand and complete an English language survey. The survey sought their views on the quality of maternity care received.

Our primary objective was to assess the construct validity and reliability of the online English language service user version of the QMNCFi. We also sought to assess the feasibility of conducting a survey of this kind in diverse contexts. Secondary objectives, which will be reported elsewhere, included evaluating the association between key variables (type and amount of maternity care received; age; parity; socio‐economic status) and quality of care scores and assessing the acceptability and feasibility of conducting evaluations of the quality of maternity care in high‐, middle‐, and low‐income settings.

### 2.1 Recruitment

In the UK and Australia, participants were recruited through service user group networks, including Maternity Voices Partnerships, maternity care‐focused Facebook groups, “X” (formerly Twitter), and maternity service user forums. We encouraged those contacted to pass the survey link onto eligible people who might be interested.

In India and Ghana, participants were recruited in two hospital clinics in New Delhi and three Child Welfare Clinics in Accra. Participants had the option of completing the survey at the point of recruitment using a computer tablet provided by the researcher or being emailed the survey link to complete later. Researchers were trained to ensure that they did not influence the mother's decision whether or not to participate in the study nor her responses if she did so. All participants were presented with an online Participant Information Sheet (PIS) which explained the purpose of the study and prompted them to ask questions if they so wished. They could do this in person in Ghana and India, or by contacting the relevant team member, whose contact details were given in the PIS (all countries).

To enable us to assess instrument test–retest reliability, participants were also asked if they were prepared to repeat the survey 2 weeks later. Those agreeing to do so were asked to supply an email address so they could be sent a repeat survey link. Participants were offered the opportunity to enter a prize draw for a voucher worth £25 (US$32); the same value was offered in each country. Participants were advised that all email addresses would be removed from the main study database and stored in a separate database until the prize draw was complete, after which their email addresses would be deleted.

### 2.2 Data Management and Analysis

The planned sample size of 120 per country was based on Mundfrom et al.'s [24] recommendations for a variables‐to‐factors ratio of about 5–6 and considering 10%–20% missing data or drop‐outs. The sample size was calculated to be large enough for factor analysis both within each country and when combined. We estimated that the sample size in each country could be reached within 2–3 months.

Exploratory factor analysis was used to assess the QMNCFi's construct validity assuming the following main constructs: Care providers; Care providers' values; Care received; Organization of care; and Management of complications (respectively, 8, 9, 13, 8, and 6 items). We used Cronbach's *α* to assess internal consistency (i.e., that questions grouped together are measuring the same construct [excellent: 0.90–1.00; good: 0.80–0.89; acceptable 0.70–0.79; borderline 0.60–0.69; poor 0.50–0.59; unacceptable < 0.50]). We also used item total correlations to determine if a global score could be developed.

A subindex score was calculated for three maternity periods (pregnancy, labor/birth, and postnatal) by summing the responses to all relevant care quality questions created for each period. These scores were expressed as a percentage (0%—perception that the care was the worst imaginable; 100%—perception that the care was the best imaginable). When combined, these subindices produce the overall “global” index score which encompassed the maternity care experience.

To evaluate whether the QMNCFi was stable over time, test–retest reliability was calculated. We recruited 55 participants who repeated the questionnaire. Requests were sent out 10–15 days following receipt of the first response, and repeat surveys received mostly within 2–4 weeks (mode 15 days; median 19; mean 23.3; SD 9.5). With an expected intraclass correlation coefficient (ICC) of 0.9, we needed 27 participants to show it is significantly different from 0.75 with 80% power. To allow for missing data, we aimed to recruit 50 participants.

## 3 Results

The survey was completed online by 540 participants (Australia 136; Ghana 131; India 153; UK 120). In India and Ghana, all respondents completed the survey on a tablet in the researcher's presence. Cronbach's Alpha results across the 13 sections showed generally very high internal consistency: six sections scored above 0.9, with another five above 0.8 and one just marginally below this (0.795). Only one section (3C) did not reach the preferred 0.7 threshold (Table 1).

**TABLE 1:** Cronbach's *α* across the QMNCFi's 13 sections.

Domains	Section	Questions about (and number of questions)	Cronbach's *α*
Care providers	1a	Care providers (*n* = 5)	0.795
	1b	Care providers' knowledge and skills (*n* = 3)	0.919
Care providers' values	2a	Respect and empowerment (*n* = 7)	0.927
	2b	Expectant management (when “care providers only intervened when this was really necessary”) (*n* = 2)	0.906
Care received	3a	Routine care (*n* = 6)	0.876
	3b	Information‐giving and advice (*n* = 2)	0.917
	3c	Support during labor (*n* = 5)	0.594
Organization of care	4a	Care location and accessibility/affordability (*n* = 2)	0.986
	4b	The maternity unit environment (*n* = 4)	0.892
	4c	Feeding the baby (*n* = 2)	0.813
Management of complications	5a	Management of minor maternal complications (*n* = 2)	0.802
	5b	Management of major maternal complications (*n* = 2)	0.879
	5c	Management of neonatal complications (*n* = 2)	0.909

**FIGURE 1:**
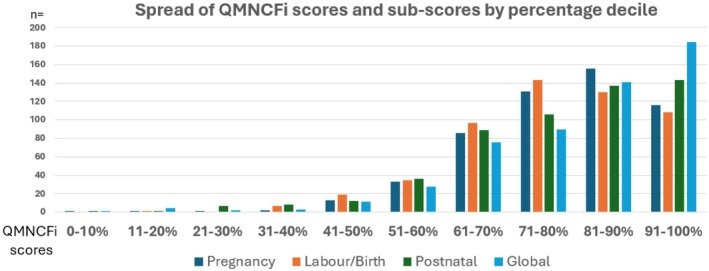
Spread of QMNCFi scores and subscores by percentage decile. [Colour figure can be viewed at wileyonlinelibrary.com]

Global QMNCFi scores ranged from 8.7% to 100% (mean 78.9%; SD 14.0%). There was a noted ceiling effect, with 116/540 participants scoring between 91% and 100% in the global score (Figure 1).

Although the average of the subindex scores was similar across Pregnancy (77.3%), Labor/Birth (78.8%) and Postnatal (81.3%) periods, scores across these time periods ranged widely in a question relating to receiving care from the same (or a known) care provider. The percentage responding “Never” was 3.1% during pregnancy, 39.3% during labor, and 14.4% in the postnatal period. There was limited international variation in average QMNCFi global scores (Australia 76.9%; Ghana 80.1%; India 84.2%; UK 72.8%).

Fifty‐five participants completed the QMNCFi a second time. Intraclass correlation coefficient results ranged from 0.657 to 0.939 across the 13 sections, indicating good test–retest reliability.

## 4 Discussion

While there are several survey tools that measure the quality of care or the quality of care organization at particular times (e.g., during labor/birth) [9, 25, 26, 27], we believe this is the first globally focused service user instrument based on evidence that assesses maternity care quality comprehensively across the whole pregnancy‐birth‐postnatal continuum. The QMNC Framework [12] was developed from the highest available evidence using a global perspective, and the international development of the QMNCFi involved experienced clinicians, researchers, and service user advocates [23]. Removing redundant items from the original 85‐item QMNCFi resulted in the current 44‐item QMNCFi.

One section (3C; Support during labor) had a lower Cronbach's *α*. We looked carefully at the relevant questions, which relate to mobility in labor, access to analgesia, physical and emotional support by the care provider, and being free to choose birth position. We reanalyzed without the emotional support question, considering that this might be an outlier, but this made no difference. The face validity of all five questions is so high that we decided we could not omit any of them. It may be that the lower value is due either to lower response rates as these questions excluded the 21% of participants (117/540) who did not go into labor, or simply to expected statistical variation in the data.

That section apart, the Cronbach's *α* scores were excellent or high. This validation study shows the QMNCFi has good internal consistency as well as good test–retest reliability for the measurement of quality care over time in diverse and complex maternity care settings.

The evaluation of maternity care is multifaceted and complex. Previous research has focused on addressing the measurement of satisfaction [28] and health‐related quality of life [29] as well as the evaluation of subjective experiences such as pain or of care provider performance [30]. While there has been an increase in the reported use of patient‐ or participant‐reported outcome measures [31, 32], there has also been some criticism of subjective evaluations due to a perceived lack of standardization and of reliability [33]. Indeed, the link between satisfaction and health care or between health care quality and outcomes is reported to be unclear or even tenuous [34, 35]. Other research in India has demonstrated widespread disrespect and abuse during labor and birth, with calls for health facilities and services to listen to women's voices [36, 37]. Similarly, research from Ghana has described the need to address mistreatment and to listen to women's voices, particularly when they experience complications from birth [38, 39]. The QMNCFi takes these concerns into consideration, overcoming many of these weaknesses and criticisms. First, its comprehensive basis has been determined by a high‐level global evidence synthesis [12]. Second, its composition has been ratified by highly experienced stakeholders [23]. Third, its psychometric properties have been confirmed in a multinational study involving low‐middle as well as high‐income countries. Additionally, the option of in‐person face‐to‐face completion (as adopted in India and Ghana) allows for the inclusion of women who are not able to read the survey form themselves, whether online or in paper form. Finally, the high rates of completion and reported acceptability and reliability show it to be a feasible method of assessing perceptions of quality care at scale over time. In another paper, we will report how we used correlation and regression analysis to identify associations between item and global scores with identified characteristics (age, parity, household income, time since the birth, and type of maternity care received).

Ensuring high‐quality care requires accurate measurement as a baseline and for benchmarking purposes. The QMNCFi results reflected a wide range of experiences, with lower scores indicating a poor perception of care quality, and higher scores indicating a positive perception of care quality. The QMNCFi was able to make discriminatory assessments, for example, over continuity of care at different time periods. As with many satisfaction measures, particularly in maternity care, the scores showed a ceiling effect. In Ghana and India, the survey was completed face‐to‐face in a clinical environment, and we cannot discount the possibility that the slightly higher scores in those countries may be partly attributable to social desirability bias.

Systematically involving communities, particularly in low‐middle income countries, promotes high‐quality care [40]. The comprehensive nature of the development of the QMNC Framework, and the involvement of multiple stakeholders in the development of the QMNCFi, reflect this inclusive approach.

We acknowledge that in India and Ghana, our study was carried out only in urban areas only. Following formal validation, there are plans to use it beyond the main cities in Ghana. As we did not ask respondents to identify their location, we cannot say what proportion of the respondents in Australia and the UK were urban or rural. An advantage of online snowball sampling is that it can reach people in more remote areas, although with an online survey we concede that digital literacy and internet access are prerequisites. However, this is not the case when completing the survey in‐person and face‐to‐face, as there is no reason why a paper‐based version could not be used.

Measures for quality care are not only required in low‐middle‐income countries. A common theme running through a recent UK parliamentary report on birth trauma was that women and birthing people felt their concerns about care went unrecognized by clinical staff and the wider health system [41]. In Australia, a recent legislative birth trauma enquiry received 4000 submissions from women [6]. The findings from these enquiries, although focused on birth trauma, demonstrate the need for a validated and comprehensive tool for service users to provide feedback to providers and maternity services on the care they received.

The QMNC Research Alliance (www.qmnc.org), an international grouping of clinicians, researchers, and service user advocates interested in developing and applying the QMNC Framework, has invited people to express an interest in using the QMNCFi, once validated. In addition to the countries included in this study, to date, there have been requests to use the QMNCFi from researchers/clinicians in 14 countries from North America, South America, Africa, Asia, the Middle East, and Europe. We are suggesting that further use, including translating the QMNCFi into other languages, should be monitored (not controlled) by the QMNC Research Alliance. To encourage networking and a robust approach, a template protocol for translation and validation in other languages, together with a “how to” video and booklet will be offered to interested researchers. The QMNCFi could be used as a “before‐and‐after” instrument as part of the evaluation of an intervention. To offer a comparison of perspectives, we intend to develop a service provider version which can be used independently or alongside the service user version.

## 5 Conclusion

Globally, research and enquiries into the quality of care indicate the need for a robust tool that effectively measures care based on the highest levels of evidence. Monitoring care quality from the service user perspective is essential if high quality care is to be maintained and poor care identified and remedied. The QMNCFi is a valid and globally relevant instrument for comprehensively assessing care quality using the concepts and characteristics of quality care identified in the evidence‐informed QMNC Framework.

## Ethics Statement

Appropriate ethics approvals were obtained in each country: United Kingdom (School Research Ethics Committee, University of Dundee [UoD/SHS/2023/007]); Ghana (Ghana Health Service Ethics Review Committee [GHS‐ERC 05/23]); India (Jamia Hamdard Institutional Ethics Committee, New Delhi [JH 5/23 (26/4/2023)]); Australia (Human Research Ethics Committee, University of Newcastle [H‐2021‐0308]).

## Conflicts of Interest

The authors declare no conflicts of interest.

## Data Availability

The data that support the findings of this study are available on request from the corresponding author. The data are not publicly available due to privacy or ethical restrictions.
